# Out-of-pocket payments, vertical equity and unmet medical needs in France: A national multicenter prospective study on lymphedema

**DOI:** 10.1371/journal.pone.0216386

**Published:** 2019-05-08

**Authors:** Gregoire Mercier, Jenica Pastor, Valerie Clément, Ulysse Rodts, Christine Moffat, Isabelle Quéré

**Affiliations:** 1 CHU de Montpellier, Montpellier, France; 2 CEPEL, UMR 5112 CNRS Université de Montpellier, Montpellier, France; 3 LAMETA, UMR5474 CNRS Université de Montpellier, Montpellier, France; 4 School of Health Sciences, University of Nottingham, Room 406 School of Health Sciences, Royal Derby Hospital, Derby, United Kingdom; 5 EA 2992 Dynamic cardiovascular inconsistencies, Université de Montpellier, UFR de Médecine de Montpellier-Nîmes, Nimes, France; University of North Texas Health Science Center, UNITED STATES

## Abstract

Out-of-pocket payments might threaten the vertical equity of financing and generate unmet medical needs. The main objective was to assess the vertical equity of outpatient out-of-pocket payments for lymphedema patients in France. Twenty-seven centres, among which 11 secondary care hospitals and 16 primary care practices participated in this prospective national multicenter study. We measured the lymphedema-specific outpatient out-of-pocket payments over 6 months. The vertical equity of out-of-pocket payments was examined using concentration curves, the Gini coefficient for income, the Kakwani index, and the Reynolds-Smolensky index. We included 231 lymphedema patients aged 7 years or more, living in metropolitan France, and being able to use Internet and email. After voluntary health insurance reimbursement, the mean out-of-pocket payment was equal to 101.4 Euros per month, mainly due to transport (32%) and medical devices (26%). Concentration curves indicated regressivity of out-of-pocket payments. Total out-of-pocket payments represented 10.1% of the income by consumption unit for the poorest quintile and 3.5% for the wealthiest (p<0.05). The Kakwani index for out-of-pocket payments was equal to -0.18.

Regarding outpatient health care, French lymphedema patients face significant and regressive out-of-pocket payments, associated with an increased risk of unmet medical needs. Such results shed light on significant socioeconomic inequalities and bring into question the current financing arrangements of outpatient health care in France.

**Trial registration:** ClinicalTrials.gov ID: NCT02988479

## Introduction

Out-of-pocket payments (OOPP) are defined as the involvement of the patient in the payment of health care services [1, 2]. They have been increasingly implemented to enhance the efficiency of health systems. OOPP might decrease moral hazard, that is to say the theoretical overconsumption of health care due to health insurance [2, 3]. OOPP can take the form of co-insurance (a proportion of the service cost), co-payment (flat-rate fees per service), and deductibles (payment of the actual cost up to a given amount before the insurance kicks in) [2].

Nonetheless, OOPP might threaten the efficiency and equity principles of health care systems. First, high OOPP are associated with unmet medical needs [4]. Second, they might create inequities. Following the principle of vertical equity in the financing of health care, individuals with unequal ability to pay should pay unequally for a given level of health [5, 6].

In France, the publicly-funded mandatory health insurance (MHI) scheme (Assurance Maladie) covers 79% of the total health care expenditure [7]. The MHI coverage is more important for inpatient care (91%) than for outpatient care (63%). In the ambulatory care sector, extra-billing is allowed for out-patient specialist medical visits, and some prescription medical devices have a free selling price above the statutory tariff. Other medical devices are either prescribed but not listed or over-the-counter and are therefore not reimbursed at all by the MHI scheme. A flat-rate copayment is applied per service (0.5 Euro for non-physician services, drugs and medical devices, 1 Euro for physician visits, medical imaging and laboratory tests, and 2 Euros for transport) up to a 50 Euros annual ceiling to all services. This ceiling does not include extra-billing or selling prices above the statutory tariff. Almost 90% of the population is covered by voluntary health insurance (VHI) that covers 14% of the health care expenditure and plays a complementary role. VHI schemes usually reimburse the MHI co-insurance fee up to 100% of the statutory tariff, and some contracts cover extra-billing. VHI are not allowed to cover the flat-rate co-payment. The poorest individuals are eligible to one of the publicly-funded complementary insurance schemes: couverture maladie universelle complementaire (CMUc) or aide a la complementaire sante (ACS). Under these schemes, patients have full coverage without co-insurance, and extra-billing is forbidden. Moreover, patients covered by the disease-based exemption scheme (affection de longue duree; ALD) are exempted from co-payments and co-insurance for health care related to a list of chronic conditions.

OOPP spending in France amounts to 8% of the health care expenditures in 2015 [7]. However, OOPP are unevenly distributed across services and the population. They are concentrated on outpatient health care services and on high-risk, high-spending individuals [8, 9]. In a 2005 study, the mean annual OOPP for outpatient care amounted to 529€ (SD = 1984€) after MHI reimbursement and to 200€ (SD 1174€) after VHI reimbursement [10]. Measuring post-VHI OOPP is particularly challenging in France due to difficult access to VHI data. In addition, the rare published studies do not account for patients’ health status or for unmet medical needs. Therefore, from a policy perspective, understanding the relationship among OOPP, income, health status and unmet medical needs is of utmost importance.

Lymphedema is a chronic condition associated with pain and swelling that poses a significant burden on patients [11]. Despite its prevalence reaching 4 per 1000 population [12], the population implications of lymphedema are not well studied [13]. According to current clinical guidelines, patients with lymphedema are primarily managed on an outpatient basis, with compression devices playing a key role on a lifelong basis. Though the evidence is scarce, several randomized controlled trials have demonstrated the efficacy of compression therapy on various outcomes (i.e. limb volume, symptoms severity, and functional status), both at the acute and maintenance phases[14–18]. Lymphedema patients in France are, hence, likely to experience high OOPP.

The aim of this study was to assess the inequalities in OOPP in patients living with lymphedema in France.

## Materials and methods

### Study design

We conducted a prospective and multicenter cohort study in France between 2014 and 2015.

The main inclusion criteria were having a lymphedema, being aged 7 years or more, living in metropolitan France, and being able to use Internet and email. Lymphedema patients were included in a convenience sample of 27 centres, among which 11 were public or private hospitals and 16 were individual physicians or physiotherapists working in a private practice. Each centre included all the consecutive patients who met the inclusion criteria during the study period. Because we anticipated a high drop-out rate due to the data collection burden, we ran a 1-month pre-inclusion phase. Only patients who agreed to participate at the end of the pre-inclusion phase were included in the study. All patients (or their legal representative) signed a written informed consent prior to inclusion. The study was approved by the French institutional review board (CPP Sud Mediterranee V; approval number 2014-A00716-41).

### Data collection

Data collection was standardized using a paper questionnaire filled in by health care professionals and an electronic questionnaire filled in by patients on a dedicated and secured Internet website. The professionals provided demographic and clinical data, and patients reported socio-economic and clinical data, health care services utilization, cost and reimbursement, and health-related quality of life measures. Health care service utilization was collected weekly. To ensure high-quality data, we accompanied patients and professionals with initial training, real-time data quality monitoring and active email and telephone communication.

We collected the following socio-economic data: employment status and category [19], monthly net post-tax household income (using an 11-category scale), VHI and special schemes entitlements (CMUc, ACS and ALD). Questions were asked about the travel distance to seek lymphedema care and subjective unmet medical needs due to travel distance or cost. A patient had a subjective unmet medical need when she declared having forgone in the previous year at least one of the lymphedema care recommended by the current French clinical guidelines [20]: medical visits, compression medical devices or physiotherapy. The monthly income by consumption unit (CU) was calculated according to the modified equivalence scale of the Organization for Economic Co-operation and Development (OECD). Consumption units are a standardized measure of the size of a household determined as follows: the first adult has a weight of 1.0, each subsequent adult has a weight of 0.7, and each person under 18 has a weight of 0.5. During the six-month follow-up, service utilization, cost and reimbursement (by MHI and by VHI, if any) were prospectively collected for the following outpatient health care services: physician visits, non-physician services, imaging and laboratory tests, drugs, prescription and over-the-counter medical devices (bandaging, multi-layer inelastic bandaging, pneumatic compression, compression garments, sticking-plaster, wound dressing), and transportation. Inpatient care was collected but not analyzed, because (i) lymphedema patients are primarily managed in the outpatient setting, and (ii) inpatient care may have generated very high amounts of OOPP for very few patients. Non-medical cost was considered as follows: housekeeping or childcare, cosmetics, clothes, shoes, and thermal therapies. Housekeeping and childcare were considered to account for the impact on family life. We included cosmetics, clothes and shoes because lymphedema often results in significant changes in skin colour and in the circumferences of limbs [11]. Finally, lymphedema patients often rely to thermal therapies in France [20]. Two types of OOPP were estimated: after MHI reimbursement and after MHI and VHI reimbursement.

Two quality of life-related measures were assessed: utility values derived from the EQ5D-3L with the French index values[21]. In the absence of French index values, capability scores were obtained from the ICECAP-A [22] using the UK index values[23].

### Missing or inconsistent data

Missing or inconsistent cost data for services with a statutory tariff were corrected using the adequate tariff, assuming no extra-billing. Regarding services without a statutory tariff (i.e., medical devices, transport and non-medical services), costs and reimbursements were checked directly with the patients by telephone.

### Sample size and statistical analyses

The sample size was calculated to estimate the annual OOPP with a given precision. We hypothesized a mean OOPP equal to 200€ and a standard deviation equal to 1150€ [10]. To have a 95% confidence interval with a total width equal to 300, 230 patients were required.

Descriptive analyses were performed using frequencies and proportions for qualitative variables and mean, median, standard deviation and range for quantitative variables. Comparative univariate analyses were performed using the Student’s or Wilcoxon test for quantitative variables and the Chi^2^ or Fisher test for qualitative variables.

To analyze the income-group differences in OOPP, we split the sample into 5 income per CU groups, each group representing one-fifth of the whole sample. Hence, the first income quintile refers to the poorest 20% and the fifth to the wealthiest 20%.

The vertical equity of OOPP was examined by describing the relationship between the ability to pay (measured by income per CU) and the amount of OOPP. We followed a standard strategy combining disaggregated and aggregated measures [24, 25]. Disaggregated evidence is presented graphically by comparing the concentration curve of income (Lorenz curve) and the concentration curve of OOPP. The Lorenz curve plots the cumulative share of the sample (from the poorest to the wealthiest) against the cumulative share of the total income they receive. The OOPP concentration curve plots the cumulative share of the sample (similarly to the Lorenz curve) against the cumulative share of OOPP. If the OOPP is strictly proportional (i.e., patients pay the same proportion of their income), then the Lorenz and OOPP concentration curves will overlap. When the poorest patients pay a higher proportion of their income (regressive payment system), then the OOPP concentration curve lies above the Lorenz curve. Aggregated measures are the Gini coefficient for income, the Kakwani index for OOPP and the Reynolds-Smolensky index. The Kakwani index K is calculated by subtracting the Gini coefficient G for income from the concentration index for OOPP, and it quantifies the extent to which OOPP depart from proportionality. The Kakwani index is negative when there are vertical inequities favouring the rich (regressive payment system) and positive when there are vertical inequities favouring the poor (progressive payment system). The Reynolds-Smolensky index measures the overall redistributive effect of OOPP on income and includes some horizontal inequities [26].

All analyses were performed using SAS software, version 9.2.

## Results

**Fig 1:**
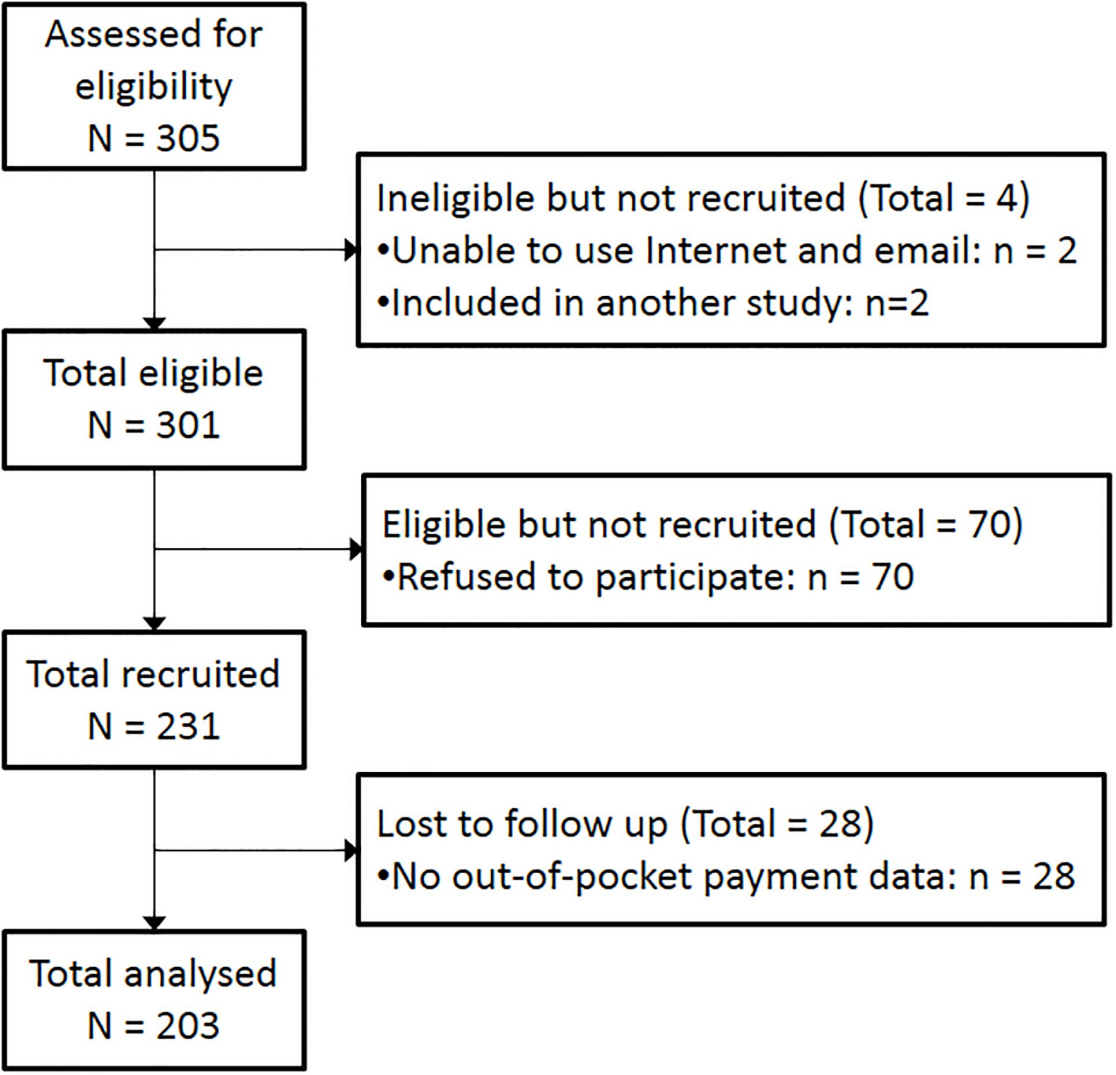
Flowchart.

A total of 305 patients were screened, 301 were eligible and participated in the pre-inclusion phase, 70 refused to participate after the pre-inclusion phase, and 231 were recruited (Fig 1). Finally, 203 patients were included in the analysis. The mean follow-up duration was 5.57 months (SD = 1.05), patients were aged 55 years on average (SD = 14.2), 6 were aged less than 18 years, and 85.7% were female (Table 1). The mean household income was 2,012 Euros per consumption unit. Regarding complementary health insurance, 97.6% of patients were covered by a VHI and 2% were entitled to a social complementary health insurance scheme.

**Table 1:** Sample characteristics.

Characteristics	Mean (SD) / N (%)
Follow-up (months)	5.6 (1.1)
Women (%)	174 (85.7%)
Age (years)	55 (14.2)
BMI (kg/m^2^)	27 (6.6)
Lymphedema type:	
• Primary	69 (34%)
• Secondary	134 (66%)
Lymphedema location:	
• Upper Limb	95 (46.8%)
• Lower Limb	105 (51.7%)
• Upper and lower limbs	3 (1.5%)
Lymphedema severity:	
• Stage 1	21 (10.4%)
• Stage 2	163 (80.3%)
• Stage 3	19 (9.3%)
Lymphedema duration:	
• from 6 months to 5 years	105 (51.7%)
• from 5 years to 10 years	28 (13.8%)
• above 10 years	70 (34.5%)
Comorbidities:	
• None	132 (65%)
• Cancer	44 (21.7%)
• Others	27 (13.3%)
Wound infection	40 (19.7%)
Hospitalization because of wound infection	30 (14.8%)
Household size (CU)	1.7 (0.7)
Household monthly income (€)	3,320 (1,861)
Monthly income per CU (€)	2,012 (1,191)
Socio-professional category:	
• Active	123 (60.6%)
• Inactive	18 (8.9%)
• Retired	62 (30.5%)
Voluntary Health Insurance (VHI) (%)	198 (97.5%)
VHI upgrade for lymphedema care	47 (23.2%)
VHI annual premium (€)	1,293 (788)
Chronic illness exemption scheme (ALD)	144 (70.9%)
Social complementary health insurance (CMUc or ACS)	4 (2%)

SD: standard deviation; BMI: body mass index; CU: consumption unit; VHI: voluntary health insurance; ALD: affection de longue duree; CMUc: couverture maladie universelle complementaire; ACS: aide a la complementaire sante.

The outpatient health care expenditure related to lymphedema amounted to 253.1 Euros (SD = 243.1) per patient per month (Table 2). Non-physician services, transport and medical devices accounted for 30%, 23% and 19% of the total expenditure, respectively. After VHI reimbursement, the mean OOPP was equal to 101.4 Euros (SD = 100) per month, mainly due to transport (32%) and medical devices (26%). Indeed, the mean OOPP for medical devices and transport amounted to 32.9 Euros (SD = 68.7) and 26.2 Euros (SD = 35.9) per month, respectively. In terms of financial burden, the mean OOPP amounted to 1,212 Euros per year or 5.4% of the annual income on average.

**Table 2:** Lymphedema-related outpatient health care expenditures and OOPP per month.

Resource	Expenditures (€)	Out-of-pocket payments (€)	
		After MHI	After VHI
Outpatient physician visits	9.9 (19.9)	1.8 (7.1)	1.1 (5.1)
Outpatient non-physician services	75.6 (77.6)	9.2 (22.9)	4.2 (18.4)
Outpatient laboratory and imaging tests	5.7 (22.3)	0.8 (5.3)	0.1 (0.6)
Pharmaceuticals	5.5 (25.4)	1.1 (4.1)	0.9 (3.7)
Medical devices	48.8 (49.7)	36.2 (41.6)	26.2 (35.9)
Transport	57.6 (112.8)	33.1 (69.1)	32.9 (68.7)
Thermal therapy	22.7 (67.7)	11.1 (36.6)	8.6 (29.7)
Others	27.5 (81.4)	27.3 (81.5)	27.3 (81.5)
**TOTAL**	**253.1 (243.1)**	**120.5 (132.1)**	**101.4 (123.1)**

Data presented are mean (standard deviation). MHI: mandatory health insurance; VHI: voluntary health insurance.

Table 3 shows the income, health status, health care expenditure, OOPP, and subjective unmet medical needs for each income quintile of the sample. The poorest patients had a poorer health status, as measured by the EQ5D-3L (0.62 for quintile 1 vs. 0.75 for quintile 5, p<0.05) and by the ICECAP-A (0.79 for quintile 1 vs. 0.90 for quintile 5, p<0.05). Although the differences are not statistically significant, there is a tendency toward lower total health care expenditure (225.3€ for quintile 1 vs. 251.4€ for quintile 5, p>0.05), lower VHI premiums (1,110€ for quintile 1 vs. 1,452€ for quintile 5, p>0.05), and lower OOPP (82.4€ for quintile 1 vs. 126.7€ for quintile 5, p>0.05). Total OOPP represented a higher share of the income by consumption unit (10.1% for quintile 1 vs. 3.5% for quintile 5, p<0.05); the same held true for OOPP due to medical devices (3.1% for quintile 1 vs. 1.1% for quintile 5, p<0.05). In total, 63 patients (31%) declared subjective unmet medical needs related to lymphedema, among which 79% were because of cost and 21% because of distance to the provider. The poorest patients declared such unmet needs more often (54% for quintile 1 vs. 17% for quintile 5, p<0.05).

**Table 3:** Distributional analysis of health status, expenditures, OOPP and unmet medical needs by income quintiles.

	Q1	Q2	Q3	Q4	Q5	p-value
Income by CU (€)	817.5 (243.3)	1,354.6 (170.3)	1,800.2 (149.1)	2,480.5 (70.2)	3,601.5 (1,516.3)	*
Outpatient health care expenditures (€)	225.3 (168.1)	240.9 (198.1)	242.9 (217.4)	306.2 (384.9)	251.4 (190.6)	
Post-VHI total OOPP (€)	82.4 (76.6)	85.3 (95.7)	108.3 (154.4)	103.9 (149.3)	126.7 (121.8)	
Post-VHI OOPP due to transport (€)	29.6 (47.3)	28.9 (52.6)	53.3 (121.6)	23.3 (39.4)	28.9 (45.4)	
Post-VHI OOPP due to medical devices (€)	25.5 (27.5)	21.8 (28.0)	16.8 (16.9)	27.9 (30.9)	39.1 (58.9)	*
Total OOPP / income ratio (%)	10.1	6.3	6.0	4.2	3.5	*
Transport OOPP / Income ratio (%)	3.6	2.1	3.0	0.9	0.8	
Medical devices OOPP / Income ratio (%)	3.1	1.6	0.9	1.1	1.1	*
Subjective unmet medical needs (n, %)	22 (54%)	13 (33%)	15 (37%)	6 (15%)	7 (17%)	*
EQ5D-3L	0.6 (0.2)	0.7 (0.3)	0.8 (0.2)	0.8 (0.2)	0.8 (0.2)	*
ICECAP-A	0.8 (0.2)	0.7 (0.2)	0.8 (0.2)	0.8 (0.2)	0.9 (0.1)	*

* P-value < 0.05

**Fig 2:**
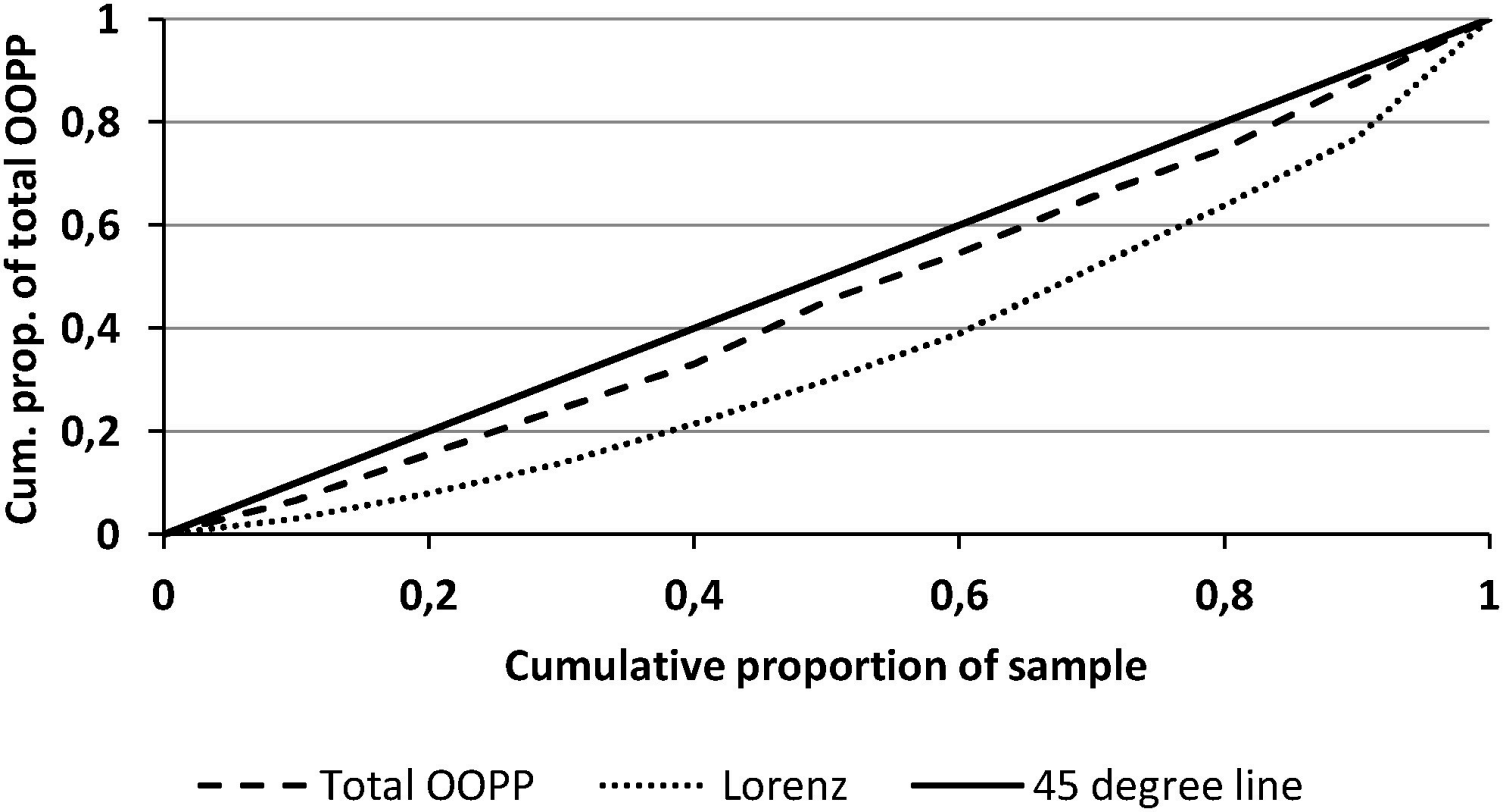
Concentration curve for total out-of-pocket payments.

Fig 2 shows the concentration curve of income per CU (Lorenz curve) and the concentration curve of total post-VHI OOPP. The concentration curve for OOPP is above the Lorenz curve and close to the 45-degree line, indicating regressivity. The Gini coefficient for income was equal to 0.29, and the concentration index for OOPP was equal to 0.11. Hence, the Kakwani index for total OOPP was equal to -0.18, denoting the regressive effect of OOPP in lymphedema patients. For instance, the poorest 20% of the sample receives less than 8% of the total income and pays 16% of the total OOPP. The Reynolds-Smolensky index was equal to -0.01.

## Discussion

In this study, French patients with lymphedema experience significant post-VHI OOPP regarding outpatient health care. Indeed, even though we focused on a single condition, the annual OOPP due to outpatient lymphedema care (101 € per month or 1,212 € per year) is 6 times higher than the average annual OOPP for outpatient care of the general population in France (200€ per year) [10]. Although the rate of OOPP as a percentage of total expenditures on health is low in France compared to other OECD countries [7], the results confirm that OOPP are high for patients with chronic diseases and are concentrated on few health care services, including prescription medical devices. This financial burden posed by lymphedema-related OOPP amounted to 5.4% of the annual income on average, which is more than five times higher than the average financial burden of the general French population, i.e. 1%[10]. This is all the more true that our study focused on a single condition.

Approximately 60% of these OOPP are due to medical devices and to transport, although they represent less than 50% of the total expenditure. This points to the central role of medical devices in the current clinical guidelines [20] and to the paucity of reference centres in France. Additionally, the high share of OOPP due to medical devices raises the issue of the current pricing policy of prescription medical devices that allows for free selling prices above the existing statutory tariffs. Almost one-third of the patients declared having forgone lymphedema-related health care because of cost or distance to seek care. This rate of subjective unmet medical needs is higher than that usually measured on the general population. Indeed, in 2012, 26% of the general French population said they had forgone medical care, but 75% of the declared unmet medical needs were related to dental care and vision care [27]. In the same study, 3% of the general population had forgone medical care because of distance, versus 12% in our lymphedema sample. On unmet medical needs, the OECD data focus on forgone medical or dental examination, and hence, the French average is lower (4.5%) [7]. Unmet medical needs are usually associated with poorer health status and lower health care utilization [4]. This holds true in our study and is consistent with published evidence from France [28].

The burden of OOPP fell unequally on different income groups, with patients from the poorest quintile paying an approximately three times larger share of their income than patients from the wealthiest quintile. This suggests that the principle of vertical equity in financing is violated [7]. The negative Kakwani index for total OOPP (-0.18) confirms the regressive role of OOPP in the income of lymphedema patients. This result is consistent with studies from other high-income countries suggesting a regressive effect of expenditures on medical devices on income in Austria [29] and in Hungary [30]. More generally, OOPP have been shown to increase inequalities in access to healthcare in Western and Eastern European countries [31]. The regressive effect of post-VHI OOPP in our lymphedema sample is a little lower than the regressive effect of post-MHI OOPP in the general population (-0.21; [32]). Both are for outpatient care only, but the population is significantly different in terms of age, gender and comorbidities. Hence, a direct comparison is challenging. In theory, such inequalities could be explained by the effect of different health statuses or preferences. Here, the poorest patients have lower total health care expenditures, poorer health status, and a higher rate of subjective unmet medical needs. Although they are in poorer health, the less-affluent patients have lower health care expenditures. This finding is in line with the published evidence from other countries with generous welfare arrangements and might be explained by a range of factors including cultural capital, psychosocial stress, material resources, and geographical segregation [33].

### Strengths and limitations

This study suffers from some limitations that should be acknowledged. First, we had to exclude 28 patients for whom data on out-of-pocket payment was missing. However, we did so to improve the data quality, and there is no argument for a systematic bias. Second, we improved the representativeness of the sample by including a wide range of public and private centres throughout the country, and by including all consecutive patients who met the inclusion criteria in each centre. However we were not able to check the representativeness because of lacking data on the characteristics of the lymphedema centres and patients in France. In addition, because we did not include patients who were unable to use the Internet and email, the oldest and most disabled patients are likely to be underrepresented in our study sample. Again, we do not believe this might challenge the main findings. Finally, our analysis is focused on ambulatory care costs and it might be argued that inpatient care could generate very high out-of-pocket costs. However, we did so because inpatient admissions would have generated high costs but very low out-of-pocket costs. Indeed, out-of-pocket costs are concentrated on ambulatory care services in France for several reasons [8, 9]: inpatient care is predominantly covered by the mandatory social insurance (91% on average, vs. 63% for ambulatory services), hospital medical devices are not billed to patients, transportation from and to hospitals are covered by the mandatory social insurance, and there is no extra-billing.

Nevertheless, this is one of the only OOPP studies in France accounting for a wide array of data. Indeed, we were able to prospectively collect socio-economic and clinical data as well as unmet medical needs and reimbursements by VHI that are not available in standard retrospective analyses. The national, multicenter and primarily outpatient-based pre-screening and screening certainly made the sample representative of the whole lymphedema population. As an example, the Gini coefficient for income in our sample (0.286) is very close to that of the French general population measured in 2013 (0.294; [34]. Finally, the data quality check process allowed us to control the impact of missing and inaccurate data.

### Policy implications

We show that the vertical equity principle is violated regarding the financing of outpatient lymphedema health care in France. Although cost-sharing has been implemented in most European countries, its ability to reduce the utilization of unnecessary health care remains unclear [2, 35]. According to the most widely accepted prevalence data[11], the total number of patients living with a lymphedema in France is equal to 268,000. Based on this figure and on our results, taking over the transportation OOPP (32.9 Euros per patient per month) would cost 105.8 Million Euros per year to the system. Similarly, eliminating the medical devices OOPP (26.2 Euros per patient per month) would cost 84.3 Million Euros per year. However, improving the vertical inequity does not imply eliminating OOPP. In France, various income- or disease-based mechanisms should control OOPP. However, they are not effective in protecting the poorest households from high levels of OOPP. Other budget-neutral or not options should be considered, such as adding a ceiling on OOPP [36] to the existing financing mechanisms, with or without removing the disease-based ALD exemption scheme [37]. Another option could be to implement a single co-payment for outpatient care with an annual ceiling, which may or may not be modified according to income alone [38] or to income and household composition [39].

As regards prescription medical devices specifically, OOPP are frequently explained by the difference between the statutory service and the selling price. Policy makers should therefore reconsider the possibility of charging a free selling price for medical devices. Likewise, the large amount of OOPP due to transport stresses the issue of geographical access to reference centres across the country. Possible levers for action include an improved distribution of reference centres across the territory and telehealth.

More generally, our results contribute to the debate about the consequences of cost-sharing in health care. Cost-sharing mechanisms have constantly been associated with reduced health care utilization and expenditures since the seminal RAND experiment [40]. But the consequences on health status are controversial: greater cost sharing is associated with reduced use of unnecessary care but also with reduction in utilization of high-value care such as long-term medications and preventive services [41, 42]. Our study suggests that patients facing out-of-pocket costs regarding ambulatory care for chronic oedema declare unmedical medical needs more often than the general population (31% vs. 26%, though figures are not directly comparable). Furthermore, the poorest patients declare more unmet medical needs, have lower total health care expenditures, have lower level of quality of life, and dedicated a higher share of their income to out-of-pocket costs. However, investigating whether these patients forgo necessary or unnecessary services was beyond the scope of our observational study and should be investigated using a natural experiment design.

### Conclusion

This work shows that regarding outpatient health care, French lymphedema patients face significant and regressive OOPP, associated with an increased risk of unmet medical needs. Such results shed light on significant socioeconomic inequalities and bring into question the current financing arrangements of outpatient health care in France.

## Supporting information

S1 File Study questionnaire. (DOC)

S2 File De-identified dataset. (XLSX)
