# Supplementary material for: Out-of-pocket payments, vertical equity and unmet medical needs in France: A national multicenter prospective study on lymphedema
Source: PLoS One. 2019 May 8;14(5):e0216386. doi: 10.1371/journal.pone.0216386 (PMC6506146; doi:10.1371/journal.pone.0216386)
Supplement: S1 File — (DOC) [file pone.0216386.s001.doc]

**TITRE DE L'ETUDE**

**"Reste à Charge des patients atteints de lymphœdème : mesure des inégalités financières d’accès aux soins. (LYMPHORAC)."**

Le cahier de recueil est organisé en trois sections :

1. **Données initiales :** recueil réalisé une seule fois, lors de l'inclusion du patient au démarrage de l'étude
2. **Suivi hebdomadaire :** recueil réalisé une fois par semaine, depuis l'inclusion de l'étude et pendant 6 mois
3. **Qualité de vie :** recueil réalisé 3 fois au total : à l'inclusion, à 3 mois et à 6 mois

Afin de simplifier le travail de recueil, la saisie des données se fera exclusivement via un site Internet dédié, par un identifiant personnel sécurisé.

# Avant d'utiliser le cahier d'observation,

# assurez-vous que le patient soit bien éligible.

# Information du patient :  Fait  Non fait

# Vérification de critères d'inclusion :  Fait  Non fait

| **Nro** | **Critères d’inclusion :** | **Oui** | **Non** |
| --- | --- | --- | --- |
| 1 | Patient majeur, présentant un lymphœdème primaire ou au cours du cancer, quel que soit le stade de la maladie et l’ancienneté du lymphœdème. |  |  |
| 2 | Patient suivi dans l’un des centres participant à l’étude Lymphorac. |  |  |
| 3 | Patient (ou son représentant légal) ayant donné son consentement éclairé |  |  |
| 4 | Patient acceptant de renseigner le montant des dépenses de santé restant à sa charge pendant 6 mois et de façon hebdomadaire. |  |  |
| 5 | Patient acceptant le recueil de ses données économiques. |  |  |
| 6 | Patient disposant d’internet et d’une adresse mail. |  |  |

***Important :***

***Si au moins un critère d'inclusion est coché "NON", le sujet ne doit pas être inclus dans l’étude.***

| **Nro** | **Critères de non inclusion :** | **Oui** | **Non** |
| --- | --- | --- | --- |
| 1 | Patient mineur. |  |  |
| 2 | Patient souffrant d’Insuffisance veineuse chronique concomitante au lymphœdème (ou autre pathologie qui nécessiterait des soins proches de ceux du lymphœdème). |  |  |
| 3 | Patient non assuré au régime de la sécurité social. |  |  |
| 4 | Patient refusant de participer à l’étude ou ne désirant pas renseigner le montant des dépenses de santé restant à sa charge pendant une période de 6 mois. |  |  |
| 5 | Patient sous tutelle ou curatelle. |  |  |

***Important :***

***Si au moins un critère de non inclusion est coché "OUI", le sujet ne doit pas être inclus dans l’étude.***

#

# Note d'information et Non-opposition lu:  Oui  Non

Si oui, date de signature: |__|__|/|__|__|/20|__|__|

Si OUI, date d'inclusion : |__|__| /|__|__| /20 |__|__|

# SECTION 1 : DONNEES INITIALES

# Données démographiques et cliniques à l'inclusion : A REMPLIR PAR LE PROFESSIONNEL SUR PAPIER

# Nom de patient : …………………………………………………………………………………….

# Prénom de patient : ………………………………………………………………………………

# Adresse mail de patient : …………………………………………@..........................................

# Nro de département de domiciliation : |__|__|

# Sexe :  Masculin  Féminin

# Âge du patient : |__|__| ans et |__|__|mois

# Localisation du lymphœdème :

#  Main  doigts avant-bras

#  Bras  épaule poitrine

#  Abdomen  région pubienne région fessière

#  Orteils  pied  cheville

#  Jambe  cuisse  Autres

# Cause du lymphœdème :

#  Primaire Secondaire

# Secondaire à un cancer Secondaire Veineuse

# Secondaire Obésité  Secondaire filariose lymphatique

# Secondaire Immobilité

#  Autres

# Sévérité du lymphœdème :

#  Stade I  Stade II Stade III

# Ancienneté du lymphœdème :

#  > 6 mois  6 mois à 1 année 1 à 2 ans

#  2 à 5 ans  5 à 10 ans  > 10

# Prise en charge thérapeutique actuelle :

#  Aucune prise en charge thérapeutique

#  médication (antibiotiques, diurétiques)

#  massage (avec drainage lymphatique)

#  kinésithérapie

#  gymnastique et mobilisation

#  conseils pour les soins de peau

#  vêtement de compression (panty)

#  port de bandages

#  bas de contention (chaussettes, bas-cuisse, collants)

#  plusieurs bas de contention

#  manchons

#  support psychologique

#  réduction/ lipoédème/ chirurgie lymphatique.

#  Autres

# Comorbidités associées :

# Cancer

#  diabète,

#  neuropathie,

#  insuffisance cardiaque/ maladie cardiaque ischémique,

#  maladie artérielle périphérique,

#  Autres.

# Pôle LYMPHORAC : _____________________________

# Données socio-économiques à l'inclusion : A REMPLIR PAR LE PATIENT (format électronique)

# Quelle est votre profession ou catégorie sociale ?

# Agriculteurs exploitants

# Artisans, commerçants et chefs d'entreprise

# Cadres et professions intellectuelles supérieures

# Professions Intermédiaires : les infirmiers, les sages-femmes ; les spécialistes de la rééducation ; les techniciens médicaux et préparateurs de pharmacie ; les spécialistes de l'intervention socio-éducative ; les animateurs socioculturels et de loisirs, les personnels enseignants de l'enseignement primaire (professeurs des écoles, instituteurs), les personnels enseignants non agrégés ou certifiés, les maîtres auxiliaires et professeurs contractuels de l'enseignement secondaire, les conseillers principaux d'éducation, surveillants, les formateurs et animateurs de formation continue, les moniteurs et éducateurs sportifs, ainsi que les sportifs professionnels.

# Employés

# Ouvriers

# Retraités

# Chômeur

# Autres personnes sans activité professionnelle. Précisez :

# Chômeur n’ayant jamais travaillé

# Elève, étudiant(e)

# Autres.

# Quelle est la catégorie sociale et profession de votre conjoint ?

# Agriculteurs exploitants

# Artisans, commerçants et chefs d'entreprise

# Cadres et professions intellectuelles supérieures

# Professions Intermédiaires : les infirmiers, les sages-femmes ; les spécialistes de la rééducation ; les techniciens médicaux et préparateurs de pharmacie ; les spécialistes de l'intervention socio-éducative ; les animateurs socioculturels et de loisirs, les personnels enseignants de l'enseignement primaire (professeurs des écoles, instituteurs), les personnels enseignants non agrégés ou certifiés, les maîtres auxiliaires et professeurs contractuels de l'enseignement secondaire, les conseillers principaux d'éducation, surveillants, les formateurs et animateurs de formation continue, les moniteurs et éducateurs sportifs, ainsi que les sportifs professionnels.

# Employés

# Ouvriers

# Retraités

# Chômeur

# Autres personnes sans activité professionnelle. Précisez :

# Chômeur n’ayant jamais travaillé

# Elève, étudiant(e)

# Autres.

# Quelle est la taille de votre ménage, y compris vous-même :

# Nombre d’adultes : /____/

# Nombre d’enfants de moins de 14 ans : /____/

# Nombre d’enfants de plus de 14 ans : /____/

# Quel est le niveau de revenu mensuel de votre ménage (y compris les salaires nets, allocations familiales, pensions et autres revenus) ?

 Moins de 450 €

 451 € à 600 €

 601 € à 750 €

 751 € à 1 000 €

 1 001 € à 1 500 €

 1 501 € à 2 000 €

 2 001 € à 3 000 €

 3 001 € à 4 500 €

 4 501 € à 6 000 €

 6 001 € à 7 500 €

 Plus de 7 500 €

# Access au traitement:

# La prise en charge médicale de votre lymphœdème, finalement, est-t-elle couverte entre la sécurité sociale et votre assurance complémentaire santé dans sa totalité?

#  Oui non

# En termes de distance: Le traitement spécialisé de votre lymphœdème est accessible par un déplacement raisonnable?

#  Oui non

# Nombre de Km?

# Etes-vous privé(e) d'accéder au traitement spécialisé de votre lymphœdème à cause de la distance/manque de transport avec fréquence?

#  Oui non

# Est-ce que vous bénéficiez de la CMU (Couverture Maladie Universelle) ?

#  Oui  non

# Est-ce que vous bénéficiez de la CMUC (Couverture Maladie Universelle Complémentaire) ?

#  Oui  non

# Est-ce que vous bénéficiez d’une assurance maladie complémentaire ou mutuelle santé ?

#  Oui  non

# Est-ce que vous bénéficiez de l'aide à l'acquisition d'une complémentaire santé (ACS) ?

#  Oui  non

# Afin d'être mieux remboursé, avez-vous souscrit un contrat plus cher?

#  Oui  non

# Votre lymphœdème a eu-t-il ou a-t-il une incidence sociale ?

#  Oui  non

# Avez-vous continué à travailler ?

#  Oui  non

# Le lymphœdème a eu-t-il une incidence sur votre salaire et l’évolution de votre carrière ?

#  Oui  non

# Si Oui, mi-temps thérapeutique déclaré :

#  Inapte au travail.

#  Reclassement.

#  Adaptation de poste

#  Invalidité.

#  Licenciement.

#

# Si déclaré en tant qu’invalidité : Etes-vous le titulaire de la pension d’invalidé :

#  Oui non

# Cela a eu-t-il un impact sur votre vie :

# Au quotidien  Oui non

# Au niveau financier  Oui non

# Perte de revenu annuel en pourcentage ? Ou en valeur absolue : ______% __________Euros

# SECTION 2 : SUIVI HEBDOMADAIRE (rempli par le patient au format électronique)

Chaque fin de semaine, nous vous demanderons de bien vouloir renseigner :

- Toutes les dépenses engagées du fait de votre lymphœdème lors de la semaine ;
- Les remboursements correspondant, au fur et à mesure ;
- L'aide dont vous avez bénéficié de la part de vos proches ;
- Les arrêts de travail éventuels.

~~Deux guides seront à votre disposition :~~

- ~~Guide de définition des soins en lien avec le lymphœdème~~
- ~~Guide d'aide au remplissage des remboursements.~~

# Suivi des dépenses et remboursements pour chacun des items :

Pour chaque item renseigné dans la section précédente, préciser les dépenses et remboursements au fur et à mesure du suivi :

- Montant total (y compris dépassement d'honoraire)
- Part remboursée par l'Assurance Maladie obligatoire
- Part remboursée par votre complémentaire santé (Assurance ou Mutuelle)

# Identification des dépenses en lien avec le lymphœdème :

NB : pour chaque catégorie, vous pouvez saisir autant d'items que nécessaire. Par exemple, si vous avez eu une consultation chez votre généraliste et une consultation chez un spécialiste la même semaine, vous créez deux entrées dans la catégorie "Consultations médicales".

# Consultations médicales :

# Date

# Type : M. généraliste, M. Rééducateur, M. Interne, Médecine vasculaire, M. Oncologue (chirurgie), Autre.

# Actes et soins paramédicaux à domicile :

# Date

# Type d'acte : Kinésithérapie, Infirmière, Autre

# Médicaments :

# Date de délivrance par la pharmacie ou le prestataire

# Nature du dispositif ou du médicament prescrit pour lymphœdème : Antidépresseur, Antalgiques, Anti-inflammatoires, Antibiotiques, autres médicaments.

# Automédication: (Lister et dater)

# Dispositifs médicaux et matériel médical :

# Date de délivrance

# Nature du dispositif, choisissez: (pictogrammes)

# Pour les membres supérieurs :


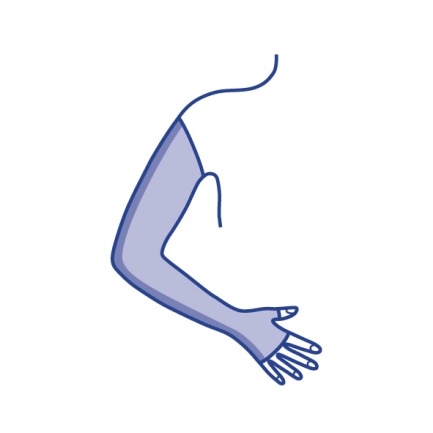

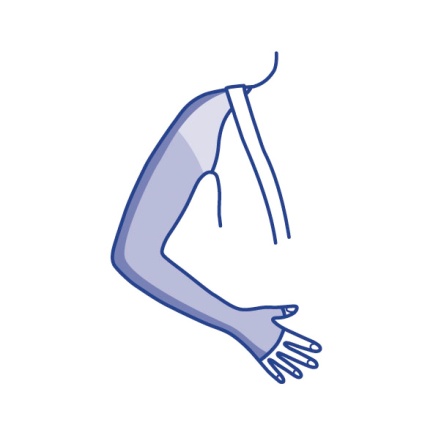

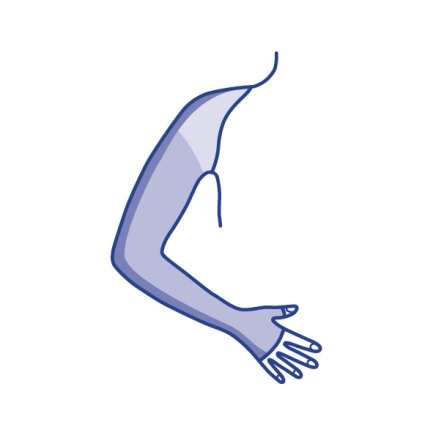

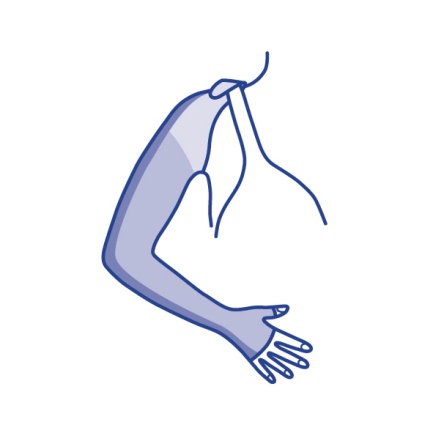

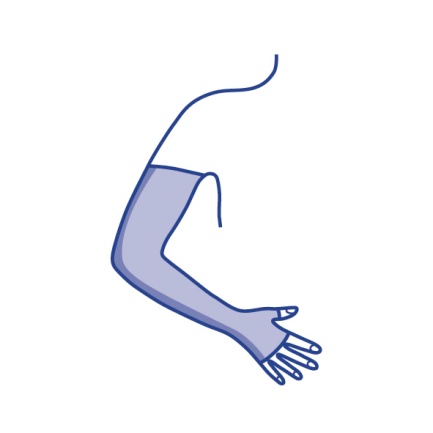

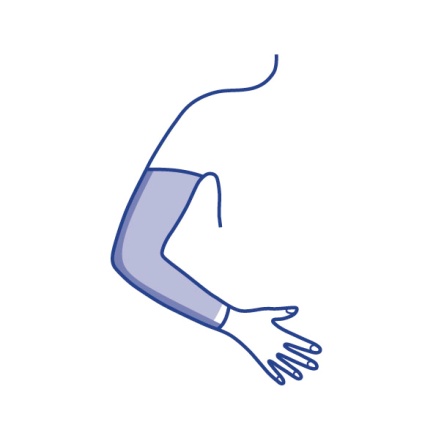

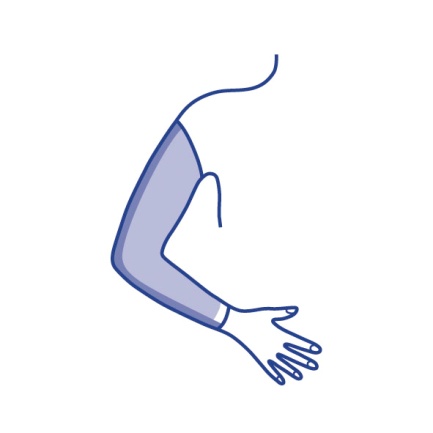

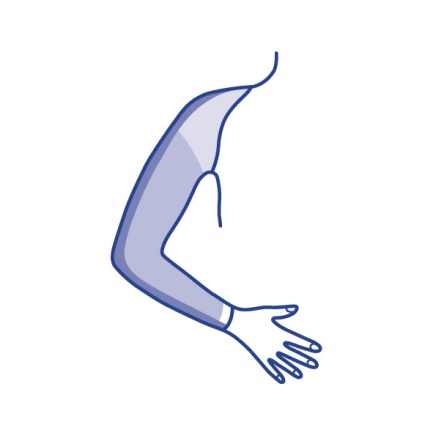

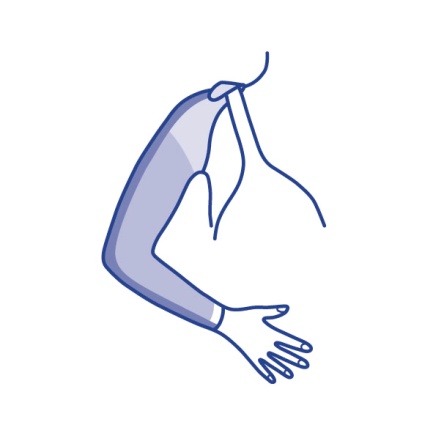

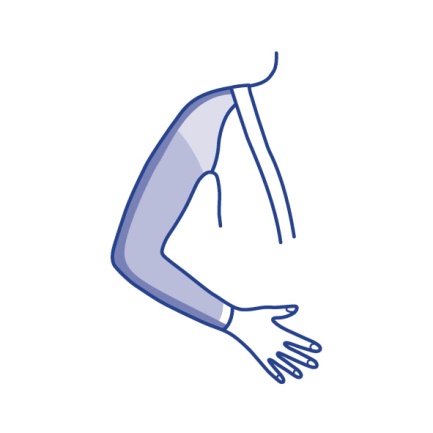

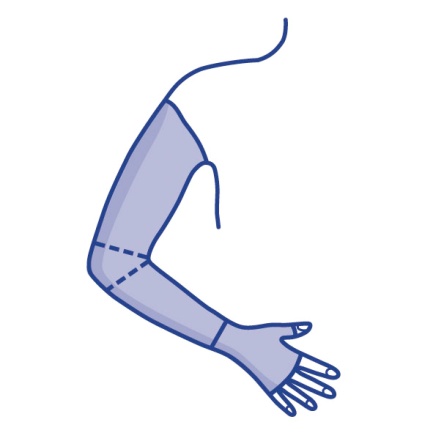

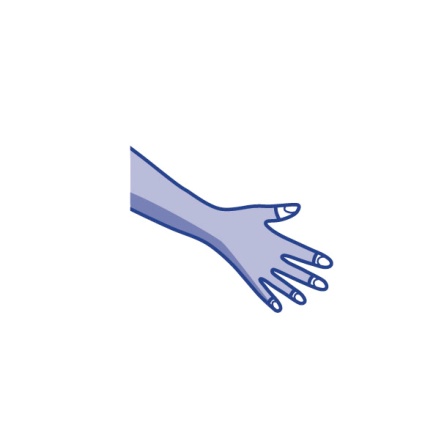

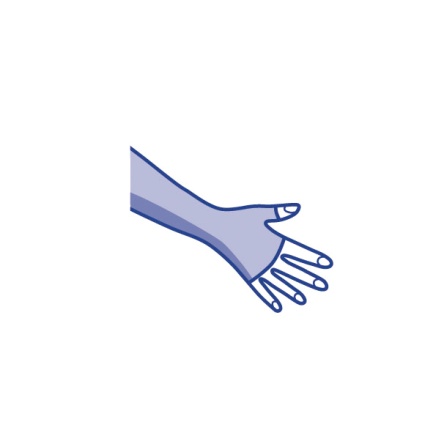

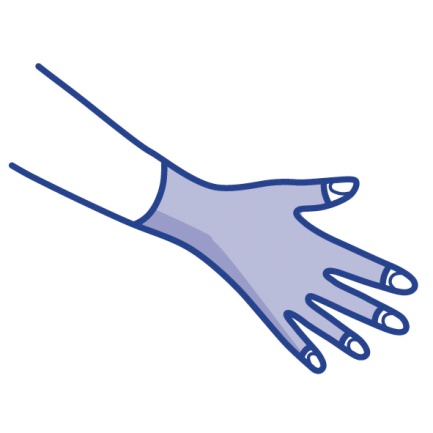


Ms1

Ms12

Ms11

Ms10

Ms9

Ms8

Ms7

Ms6

Ms5

Ms4

Ms3

Ms2

Ms13

Ms14

# Pour les membres inférieurs :


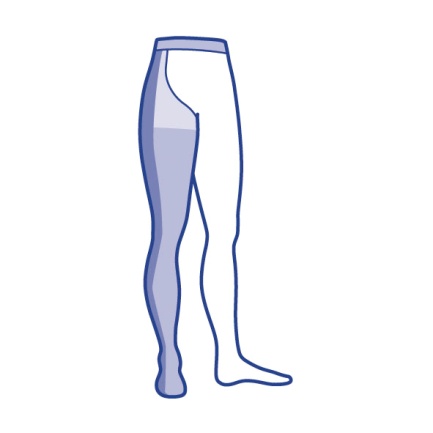

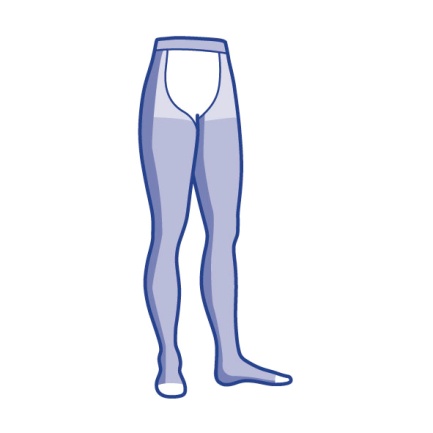

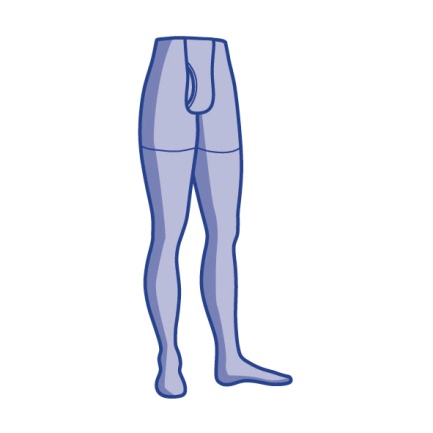

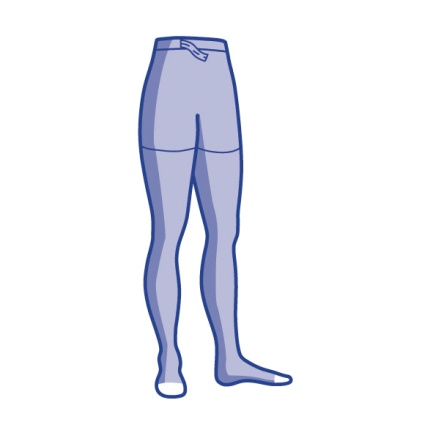

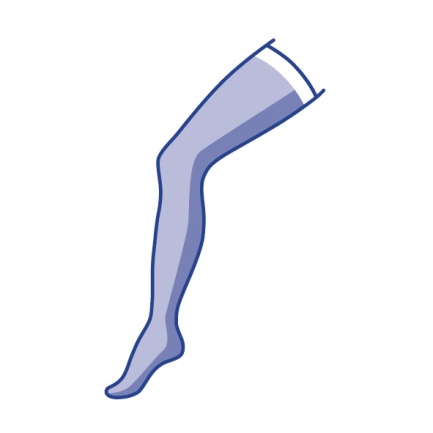

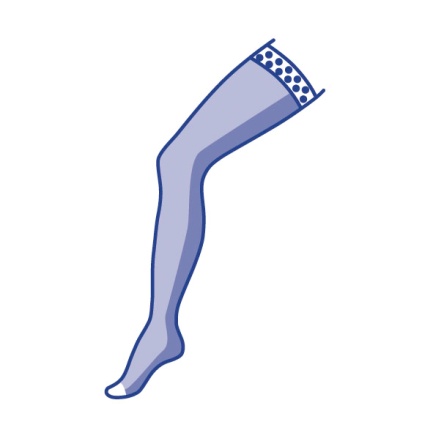

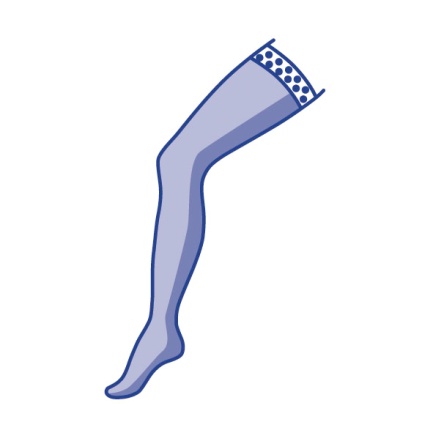

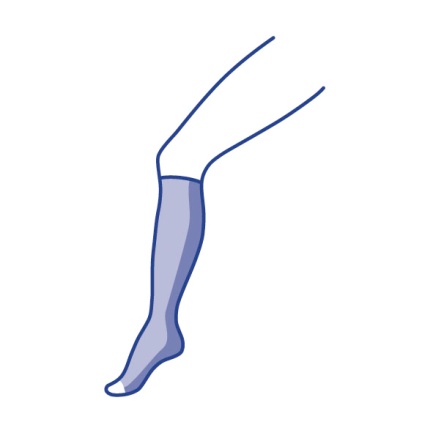

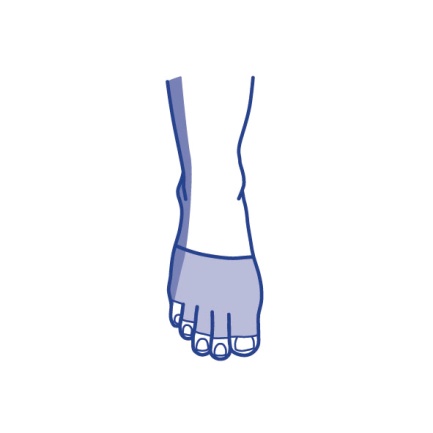

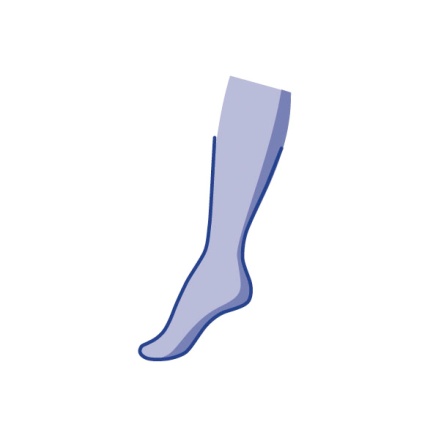

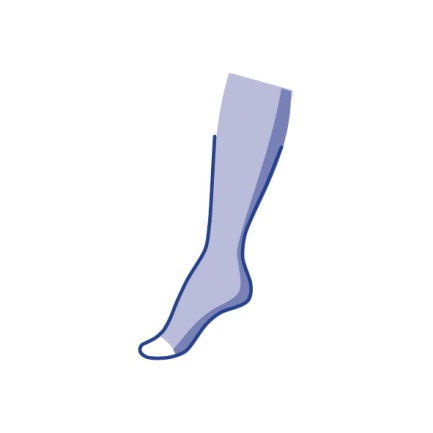


Mi1

Mi9

Mi8

Mi7

Mi6

Mi5

Mi4

Mi3

Mi2

Mi11

Mi10

# Autres dispositifs pour les membres supérieurs

# Autres dispositifs pour les membres inférieurs

# Crèmes et autres produits cosmétiques:

# Date de délivrance par la pharmacie ou le prestataire

# Cures thermales :

# Centre

# Ville

# Date de début de la cure thermale

# Date de fin de la cure thermale

# Transports médicaux :

# Date

# Type de transport : Taxi, VSL, ambulance, véhicule personnel, transports en commun, autre

# Examens de radiologie et biologie médicale :

# Date

# Type d'acte: Radiologie, biologie, échographie, écho doppler, scintigraphie, autre

# Hospitalisations :

# Date de début d'hospitalisation

# Date de fin d'hospitalisation

# Etablissement : CHU, CH, Clinique, autre précisez

# Service : médical, chirurgical, SSR

# Motif : (préciser liste)

# Embauche d’une femme de ménage ou d’une personne assurant la garde des enfants :

# Durée hebdomadaire

# Autres dépenses non médicales (ex : 2 paires des chaussures)

# Soutien de votre entourage dans les tâches de la vie domestique:

# Durée hebdomadaire

# En cas d’arrêt de travail :

# Durée en jours.

# SECTION 3 : QUALITE DE VIE A 0, 3 ET 6 MOIS. (Rempli par le patient, format électronique)

# Questionnaire EQ-5D :

# Veuillez indiquer, pour chacune des rubriques suivantes, l’affirmation qui décrit le mieux votre état de santé aujourd’hui, en cochant la case appropriée.

# Mobilité

# Je n’ai aucun problème pour me déplacer à pied 

# J’ai des problèmes pour me déplacer à pied 

# Je suis obligé(e) de rester alité(e) 

# Autonomie de la personne

# Je n’ai aucun problème pour prendre soin de moi 

# J’ai des problèmes pour me laver ou m’habiller tout(e) seul(e) 

# Je suis incapable de me laver ou de m’habiller tout(e) seul(e) 

# Activités courantes (exemples : travail, études, travaux domestiques, activités familiales ou loisirs)

# Je n’ai aucun problème pour accomplir mes activités courantes 

# J’ai des problèmes pour accomplir mes activités courantes 

# Je suis incapable d’accomplir mes activités courantes 

# Douleurs/gêne

# Je n’ai ni douleurs ni gêne 

# J’ai des douleurs ou une gêne modérée(s) 

# J’ai des douleurs ou une gêne extrême(s) 

# Anxiété/Dépression

# Je ne suis ni anxieux (se) ni déprimé(e) 

# Je suis modérément anxieux (se) ou déprimé(e) 

# Je suis extrêmement anxieux (se) ou déprimé(e) 

Pour vous aider à indiquer dans quelle mesure tel ou tel état de santé est bon ou mauvais nous avons tracé une échelle graduée (comme celle d’un thermomètre) sur laquelle 100 correspond au meilleur état de santé que vous puissiez imaginer et 0 au pire état de santé que vous puissiez imaginer.

Nous aimerions que vous indiquiez sur cette échelle où vous situez votre état de santé aujourd’hui. Pour cela, veuillez tracer une ligne allant de l’encadré ci-dessous à l’endroit qui, sur l’échelle, correspond à votre état de santé aujourd’hui.

Votre état de santé aujourd’hui

# Questionnaire de Satisfaction de Vie

# Sur une échelle de 0 à 10 – où 0 représente “pas du tout satisfait” et 10 représente “complètement satisfait » – indiquez quelle est votre satisfaction globale par rapport à votre vie :

 0

 1

 2

 3

 4

 5

 6

 7

 8

 9

 10

# Indiquer s’il vous plaît quelle phrase traduit le mieux votre qualité de vie globale en ce moment en cochant la case correspondante pour chacune des cinq dimensions :

# Sentiment de stabilité et de sécurité

| Je suis capable de me sentir à l’aise et en sécurité dans**tous**les domaines de ma vie |  |
| --- | --- |
| Je suis capable de me sentir à l’aise et en sécurité dans de **nombreux** domaines de ma vie |  |
| Je suis capable de me sentir à l’aise et en sécurité dans **quelques** domaines de ma vie |  |
| Je suis capable de me sentir à l’aise et en sécurité dans **aucun** domaine de ma vie |  |

# Amour, amitié et soutien

| Je peux avoir **beaucoup** d'amour, d'amitié et de soutien |  |
| --- | --- |
| Je peux avoir **assez** d'amour, d'amitié et de soutien |  |
| Je peux avoir **un peu** d'amour, d'amitié et de soutien |  |
| Je **ne peux pas avoir**d'amour, d'amitié et de soutien |  |

# Sentiment d’indépendance

| Je suis capable d'être **complètement**indépendant(e) |  |
| --- | --- |
| Je suis capable d'être indépendant(e) dans **beaucoup** de choses |  |
| Je suis capable d'être indépendant(e) dans un **certain** nombre de choses |  |
| Je suis **incapable** d'être indépendant(e) |  |

# Réalisation et progrès

| Je peux réussir et progresser dans**tous** les aspects de ma vie |  |
| --- | --- |
| Je peux réussir et progresser dans **plusieurs** aspects de ma vie |  |
| Je peux réussir et progresser dans**quelques**aspects de ma vie |  |
| Je suis**incapable** de réussir et de progresser quel que soit le domaine de ma vie |  |

# Joie de vivre et plaisir

| Je peux avoir **beaucoup**de joie et de plaisir dans ma vie |  |
| --- | --- |
| Je peux avoir **assez**de joie et de plaisir dans ma vie |  |
| Je peux avoir **un peu** de joie et de plaisir dans ma vie |  |
| Je **ne peux pas** avoir de joie et de plaisir dans ma vie |  |

Veuillez s’il vous plait vous assurer de n’avoir coché qu’une seule case pour chacune des cinq dimensions

# Questionnaire, Outil de mesure de la qualité de vie en cas de lymphœdème aux bras ou jambes

Ce questionnaire a été conçu et validé pour mesurer la qualité de vie des patients souffrant d'un œdème chronique ou un lymphœdème à un bras ou aux deux et à une jambe ou aux deux.

# LYMQOL ARM (LYMQOL BRAS) :

# Pour chacune des questions, veuillez cocher la case qui décrit le mieux ce que vous ressentez. Si certains de ces points ne vous sont pas applicables, veuillez écrire s.o. dans les cases correspondantes.

| **(Q1)** Dans quelle mesure le gonflement de votre bras affecte les activités quotidiennes suivantes ? | **Pas du tout** | **Un peu** | **Passa**  **blement** | **Beaucoup** |
| --- | --- | --- | --- | --- |
| a) activités professionnelles |  |  |  |  |
| b) travaux ménagers |  |  |  |  |
| c) peigner les cheveux |  |  |  |  |
| d) s'habiller |  |  |  |  |
| e) écrire |  |  |  |  |
| f) manger |  |  |  |  |
| g) se laver |  |  |  |  |
| h) se brosser les dents |  |  |  |  |

**(Q2)** Dans quelle mesure affecte-t-il vos loisirs et votre vie sociale ?

|  |  |  |  |
| --- | --- | --- | --- |

Veuillez en donner des exemples .......................................................................................................................................................................................................................................................................................................

|  | **Pas du tout** | **Un peu** | **Passa**  **blement** | **Beaucoup** |
| --- | --- | --- | --- | --- |
| **(Q3)** Etes-vous dépendant d'autres personnes? |  |  |  |  |
| **(Q4)** Estimez-vous que le gonflement affecte votre apparence ? |  |  |  |  |
| **(Q5)** Avez-vous de difficultés à trouver des vêtements qui vous conviennent ? |  |  |  |  |
| **(Q6)** Avez-vous de difficultés à trouver des  vêtements que vous aimeriez mettre ? |  |  |  |  |
| **(Q7)** Est-ce que le gonflement affecte la façon dont vous vous percevez ? |  |  |  |  |
| **(Q8)** Est-ce qu'il affecte vos relations avec les autres ? |  |  |  |  |
| **(Q9)** Est-ce que votre lymphœdème vous fait mal ? |  |  |  |  |
| **(Q10)** Est-ce que vous sentez un engourdissement de votre bras enflé ? |  |  |  |  |
| **(Q11)** Est-ce que vous avez des fourmillements ou des picotements dans votre bras enflé ? |  |  |  |  |
| **(Q12)** Est-ce que votre bras enflé est faible ? |  |  |  |  |
| **(Q13)** Est-ce que votre bras enflé est lourd ? |  |  |  |  |
| **(Q14)** Est-ce que vous vous sentez fatigué ? |  |  |  |  |

| La semaine passée … | **Pas du tout** | **Un peu** | **Passa**  **blement** | **Beaucoup** |
| --- | --- | --- | --- | --- |
| **(Q15)** Avez-vous eu des difficultés à dormir ? |  |  |  |  |
| **(Q16)** Avez-vous eu des difficultés pour vous  concentrer, par exemple pour lire ? |  |  |  |  |
| **(Q17)** Est-ce que vous vous êtes senti tendu ? |  |  |  |  |
| **(Q18)** Est-ce que vous vous êtes senti inquiet ? |  |  |  |  |
| **(Q19)** Est-ce que vous vous êtes senti irritable ? |  |  |  |  |
| **(Q20)** Est-ce que vous vous êtes senti déprimé ? |  |  |  |  |

**(Q21)** Dans l'ensemble, comment jugez-vous votre qualité de vie actuelle ?

Veuillez indiquer votre note dans l'échelle suivante:

0 1 2 3 4 5 6 7 8 9 10

**Faible Excellente**

| Les questions du 15 à 20 ont été reproduites avec la permission de l'EORTC. Ces questions ne forment qu'une partie du questionnaire QLC-C30 |
| --- |

Copyright novembre 2007 Réf ARM V II

Tous droits réservés. Ce document peut être reproduit ou utilisé librement à condition que cette déclaration de copyright soit laissée intacte, que la source soit indiquée, que l'utilisateur 􀁖􀂶enregistre et qu'aucune modification ne soit effectuée sans la permission de l'auteur. Les demandes d'autorisation et d'enregistrement doivent être transmises par écrit au Dr Vaughan Keeley, consultant en médecine palliative, Nightingale Macmillan Unit, 117A London Road, Derby DE1 2QS.

# LYMQOL LEG (LYMQOL JAMBES)

Pour chacune des questions, veuillez cocher la case qui décrit le mieux ce que vous ressentez. Si certains de ces points ne vous sont pas applicables, veuillez écrire s.o. dans les cases correspondantes.

| **(Q1)** Dans quelle mesure le gonflement de votre jambe affecte les activités suivantes ? | **Pas du tout** | **Un peu** | **Passa**  **blement** | **Beaucoup** |
| --- | --- | --- | --- | --- |
| a) la marche |  |  |  |  |
| b) votre capacité à vous pencher, par exemple à lacer vos chaussures ou à couper les ongles des orteils |  |  |  |  |
| c) votre capacité à vous tenir debout |  |  |  |  |
| d) votre capacité à vous lever d'une chaise |  |  |  |  |
| e) vos activités professionnelles |  |  |  |  |
| f) votre capacité à faire les travaux ménagers |  |  |  |  |

**(Q2) Est-ce que ce gonflement affecte vos loisirs ou votre vie sociale ?**

|  |  |  |  |
| --- | --- | --- | --- |

Veuillez en donner des exemples .......................................................................................................................................................................................................................................................................................................

|  | **Pas du tout** | **Un peu** | **Passa**  **blement** | **Beaucoup** |
| --- | --- | --- | --- | --- |
| **(Q3)** Etes-vous dépendant d'autres personnes ? |  |  |  |  |
| **(Q4)** Estimez-vous que le gonflement affecte votre apparence ? |  |  |  |  |
| **(Q5)** Avez-vous des difficultés à trouver des vêtements qui conviennent ? |  |  |  |  |
| **(Q6)** Avez-vous des difficultés à trouver des vêtements que vous aimeriez mettre ? |  |  |  |  |
| **(Q7)** Avez-vous des difficultés à trouver des chaussures qui vous vont ? |  |  |  |  |
| **(Q8)** Avez-vous des difficultés à trouver des chaussettes / bas / collants qui vous vont ? |  |  |  |  |
| **(Q9)** Est-ce que le gonflement affecte la façon dont vous vous percevez ? |  |  |  |  |
| **(Q10)** Est-ce qu'il affecte vos relations avec les autres ? |  |  |  |  |
| **(Q11)** Est-ce que votre lymphœdème vous fait mal ? |  |  |  |  |
| **(Q12)** Est-ce que vous sentez un engourdissement de votre/vos jambe(s) enflée(s) ? |  |  |  |  |
| **(Q13)** Est-ce que vous avez des fourmillements ou des picotements dans votre/vos jambe(s) enflée(s) ? |  |  |  |  |
| **(Q14)** Est-ce que votre/vos jambe(s) enflée(s) est/sont faible(s) ? |  |  |  |  |
| **(Q15)** Est-ce que votre/vos jambe(s) enflée(s) est/sont lourde(s) ? |  |  |  |  |

| **La semaine passée…** | **Pas du tout** | **Un peu** | **Passa**  **blement** | **Beaucoup** |
| --- | --- | --- | --- | --- |
| **(Q16)** Avez-vous eu des difficultés à dormir ? |  |  |  |  |
| **(Q17)** Avez-vous eu des difficultés pour vous  concentrer, par exemple pour lire ? |  |  |  |  |
| **(Q18)** Est-ce que vous vous êtes senti tendu ? |  |  |  |  |
| **(Q19)** Est-ce que vous vous êtes senti inquiet ? |  |  |  |  |
| **(Q20)** Est-ce que vous vous êtes senti irritable ? |  |  |  |  |
| **(Q21)** Est-ce que vous vous êtes senti déprimé ? |  |  |  |  |

**(Q22)** Dans l'ensemble, comment jugez-vous votre qualité de vie actuelle ?

Veuillez indiquer votre note dans l'échelle suivante:

0 1 2 3 4 5 6 7 8 9 10

**Faible Excellente**

| Les questions du 15 à 20 ont été reproduites avec la permission de l'EORTC. Ces questions ne forment qu'une partie du questionnaire QLC-C30 |
| --- |

Copyright novembre 2007 Réf ARM V II

Tous droits réservés. Ce document peut être reproduit ou utilisé librement à condition que cette déclaration de copyright soit laissée intacte, que la source soit indiquée, que l'utilisateur 􀁖􀂶enregistre et qu'aucune modification ne soit effectuée sans la permission de l'auteur. Les demandes d'autorisation et d'enregistrement doivent être transmises par écrit au Dr Vaughan Keeley, consultant en médecine palliative, Nightingale Macmillan Unit, 117A London Road, Derby DE1 2QS.

# Control subjectif du gonflement: A votre avis, le control du gonflement est bon?

#  Oui no Vous ne savez pas

# Date de dernier renouvellement de votre manchon : jj/mm/aaaa, ou

# Date de dernier renouvellement de votre bas : jj/mm/aaaa

English Version

| **Date:**  Day Month Year | Core Tool | | | | **Page 1**  **Site Number**  **Patient Number** |
| --- | --- | --- | --- | --- | --- |
| **Facility Number**  **Type of Facility (check one)**   | General practitioner |  | Nursing home | |  | | --- | --- | --- | --- | --- | | Community nursing / Home care service | | Elderly care residential home | | | | Acute hospital (check type of patient)  In-patient Out-patient | | Specialist Lymphoedema service  Other (specify) ­­­­­­­­­­­­­­­­­___________________*________* |  | | | | | | | |
|  | | | | | |
| **Demographics LYMPHORAC I.1.4. et I.1.5.** | | | | | |
| Gender Male  Female | | | Age | | |
| Level of Obesity (check one) | | | | | |
| Under weight Normal weight Obese Morbidly obese | | | | | |
| Mobility | | | | | |
| **Lower body** (check one) Bed boundChair bound Walks with aid Walks unaided | | **Upper body** (check one) Full range of movementLimited range of movement No function | | | |
| Relevant Co-morbidities (check all that apply) LYMPHORAC I.1.11. | | | | | |
| Diabetes MellitusNeurological disorder | | Heart failure / Ischaemic heart diseasePeripheral arterial disease | | | |
|  | | | | | |
| Classification of Lymphoedema (check one) LYMPHORAC I.1.7. | | | | | |
| Primary Secondary If Secondary, swelling due to:(check one) Cancer Non Cancer  If Non Cancer:(check one) Venous Immobility Obesity Lymphatic Filariasis Other (specify) ___________________ | | | | | |
|  | | | | | |
| **Lymphoedema History** (check one) **LYMPHORAC I.1.9.** | | | | | |
| **Estimate the duration of the lymphoedema:** <6 months 6 months – 1 year 1-2 years 2-5 years 5-10 years >10 years | | | | | |
| **Cellulitis** | | | | | |
| Has the patient ever had cellulitis? Yes No | | | |  | |
| **In the past year, has the patient had an acute infection in the affected areas due to the swelling?**  Yes No If yes, How many times: | | | | | |
| **In the past year, has the patient been admitted to hospital as a result of this infection?**  Yes No If yes, How many times: | | | | | |

|  |  | **Core Tool Page 2** |
| --- | --- | --- |

| **Main Categories of Treatment within Complex Decongestive Therapy** (check all that apply) **LYMPHORAC I.1.10.** | | | | | | | |
| --- | --- | --- | --- | --- | --- | --- | --- |
| None | | Physiotherapy (not massage) | | | Debulking / lipoedema / lymphatic surgery | | |
| Skin care advice | | Compression garment | | | Exercise advice | | |
| Wound dressing | | Multilayer bandage | | | Cellulitis advice | | |
| Antibiotic | | Compression wrap | | | Psychological support | | |
| Massage (including  lymphatic drainage) | | Pneumatic compression pumps | | | Other (specify) _________________________ | | |
|  | | | | | | | |
| Site of Swelling (check all that apply) LYMPHORAC I.1.6. | | | | | | | |
| Head  Face  Eyes  Lips  Cheek  Tongue  Neck | Vulva  Scrotum  Penis | | L fingers  L hand  L lower arm  L upper arm  L shoulder  L upper chest/breast | L toes  L foot  L lower leg  L upper leg  L buttocks  L abdomen | | R fingers  R hand  R lower arm  R upper arm  R shoulder  R upper chest/breast | R toes  R foot  R lower leg  R upper leg  R buttocks  R abdomen |

| Wound Area |
| --- |
| Does the patient have a wound? Yes NoIf Yes, (check all that apply)Site of wound:Arm/hand Leg Foot/ankle Head/neck Sacrum/buttocks Abdomen BackType of wound:Leg/foot ulcer Pressure ulcer Surgical wound (closed) Dehisced wound BurnOther (specify) ______________________________________________________________________________________ |

| **Access to Treatment** |  |  |  |
| --- | --- | --- | --- |
|  | **Yes** | **No** | **NA** |
| 1. Is this patient’s entire treatment for lymphoedema/chronic oedema available free within the healthcare system or covered by health insurance? |  |  |  |
| 1. If this patient’s treatment for lymphoedema/chronic oedema is not free within the healthcare system or not covered by health insurance are they able to pay for care? |  |  |  |
| 1. Is specialist lymphoedema treatment available for this patient within a reasonable travelling distance for this patient? |  |  |  |
| 1. Does the distance/ lack of transport arrangements deter/prevent this patient from accessing specialist treatment? |  |  |  |
| 1. Is this patient’s lymphoedema/wound preventing discharge from hospital into the community? |  |  |  |
| 1. Is this patient’s lymphoedema/wound the main reason for them remaining in a long-term care facility? |  |  |  |
| 1. Is this patient’s lymphoedema/wound the main reason for them remaining in long-term home care? |  |  |  |
|  |  |  |  |
| **Subjective Control of Swelling** |  |  |  |
| In your opinion, is the swelling well controlled? Yes No Don’t know | | | |

| **Date:**  Day Month Year | Demographic & Disability Tool | **Page 1**  **Site Number**  **Patient Number** |
| --- | --- | --- |
|  | | |
| 1. Personal History | | |
| Who do you share your house with?No one/live alone Partner/spouse Other relative Friend Other (specify) _______________Which best describes your living accommodation?Owner occupier Public rented Privately rentedNursing home Hospital Supported living accommodationIs there a car or other vehicle normally available for you or your household to use? Yes NoAt present are you: (check all that apply)Employed full-time Unemployed looking for work Looking after the houseEmployed part-time Not working due to illness Full or part time education or trainingRetired Other (specify) ____________________________Are you the main provider for your family? Yes NoWhat age did you leave full time education? No full time educationWhat is your highest educational qualification? (check one)None School certificate/diploma University diploma/degreeMasters degree Doctorate | | |
|  | | |
| **2. Daily Activities Affected by Swelling - to what extent does the swelling affect your daily activities?** | | |
| I have had to change my job or education/training. Yes No | | |
| If yes, Did this lead to a reduced income from work? Yes No | | |
| I have had to stop work or education/training. Yes No | | |
| If yes, Approximately how many years ago did this occur? | | |
| If yes, Did this lead to a reduced income from work? Yes No | | |
| My family income has been affected because of it. Yes No | | |

| **Date:**  Day Month Year | | **WHODAS 2.0**  **WORLD HEALTH ORGANIZATION DISABILITY ASSESSMENT SCHEDULE 2.0** | | | **Page 2**  **Site Number**  **Patient Number** | | | |
| --- | --- | --- | --- | --- | --- | --- | --- | --- |
| **12-item version, self-administered**  This questionnaire asks about difficulties due to health conditions. Health conditions include diseases or illnesses, other health problems that may be short or long lasting, injuries, mental or emotional problems, and problems with alcohol or drugs.  Think back over the past 30 days and answer these questions, thinking about how much difficulty you had doing the following activities. For each question, please circle only one response. | | | | | | | | |
| **1. In the past 30 days, how much difficulty did you have in:** | | | | | | | | |
|  |  | | None | Mild | | Moderate | Severe | Extreme or cannot do |
| S1 | Standing for long periods such as 30 minutes? | |  |  | |  |  |  |
| S2 | Taking care of your household responsibilities? | |  |  | |  |  |  |
| S3 | Learning a new task, for example, learning how to get to a new place? | |  |  | |  |  |  |
| S4 | How much of a problem did you have joining in community activities (for example, festivities, religious or other activities) in the same way as anyone else can? | |  |  | |  |  |  |
| S5 | How much have you been emotionally affected by your health problems? | |  |  | |  |  |  |

| **2. In the past 30 days, how much difficulty did you have in:** | | | | | | |
| --- | --- | --- | --- | --- | --- | --- |
|  |  | None | Mild | Moderate | Severe | Extreme or cannot do |
| S6 | Concentrating on doing something for ten minutes? |  |  |  |  |  |
| S7 | Walking a long distance such as a kilometre (or equivalent)? |  |  |  |  |  |
| S8 | Washing your whole body? |  |  |  |  |  |
| S9 | Getting dressed? |  |  |  |  |  |
| S10 | Dealing with people you do not know? |  |  |  |  |  |
| S11 | Maintaining a friendship? |  |  |  |  |  |
| S12 | Your day-to-day work? |  |  |  |  |  |

| H1 | Overall, in the past 30 days, how many days were these difficulties present? |  |
| --- | --- | --- |
| H2 | In the past 30 days, for how many days were you totally unable to carry out your usual activities or work because of any health condition? |  |
| H3 | In the past 30 days, not counting the days that you were totally unable, for how many days did you cut back or reduce your usual activities or work because of any health condition? |  |

| **Date:**  Day Month Year | Quality of Life Tool **EQ-5D** | **Page 1**  **Site Number**  **Patient Number** | |
| --- | --- | --- | --- |
| Complete both the EQ-5D and LYMQOL tools for both upper and lower limbs.  Whenever possible, the patient should complete each instrument for himself or herself.  Please indicate what statements best describe your own health state today. | | | |
| **1. Mobility (check one)**  **I have no problems in walking about**  **I have some problems walking about**  **I am confined to a bed** | | | 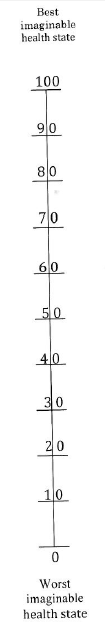 |
| **2. Self Care (check one)**  **I have no problems with self care**  **I have some problems washing or dressing myself**  **I am unable to wash or dress myself** | | |
| **3. Usual Activities: e.g. work, study, housework, family or leisure activities (check one)**  **I have no problems with performing my usual activities**  **I have some problems with performing my usual activities**  **I am unable to perform my usual activities** | | |
| **4. Pain / Discomfort (check one)**  **I have no pain or discomfort**  **I have some moderate pain or discomfort**  **I have extreme pain or discomfort** | | |
| **5. Anxiety / Depression (check one)**  **I am not anxious or depressed**  **I am moderately anxious or depressed**  **I am extremely anxious or depressed** | | |
| **6. To help people say how good or bad a health state is we have drawn a scale (rather like a thermometer) on which the best state you can imagine is marked 100 and the worst state you can imagine is marked 0.**  **We would like you to indicate on this scale how good or bad your own health is today, in your opinion. Please do this by drawing a line in the box to the right to whichever point on the scale indicates how good or bad your health state is today.** | | |

**Intentionally left blank**

| **Date:**  Day Month Year | Quality of Life Tool **LYMQOL – Upper Limb** | **Page 1**  **Site Number**  **Patient Number** |
| --- | --- | --- |

**Do you have swelling in your upper limb?**  Yes No If No, go to page 2

| **7. How much does your swollen arm affect the following daily activities** (Check one that best applies) | | | | |
| --- | --- | --- | --- | --- |
|  | Not at all | A little | Quite a bit | A lot |
| Your occupation |  |  |  |  |
| Your housework |  |  |  |  |
| Combing your hair |  |  |  |  |
| Dressing yourself |  |  |  |  |
| Your writing |  |  |  |  |
| Your eating |  |  |  |  |
| Washing yourself |  |  |  |  |
| Cleaning your teeth |  |  |  |  |

| **8.** | | | | |
| --- | --- | --- | --- | --- |
|  | Not at all | A little | Quite a bit | A lot |
| How much does it affect your leisure activities/social life? |  |  |  |  |
| How much do you have to depend on other people? |  |  |  |  |
| How much do you feel the swelling affects your appearance? |  |  |  |  |
| How much difficulty do you have finding clothes to fit? |  |  |  |  |
| How much difficulty do you have finding clothes you would like to wear? |  |  |  |  |
| Does the swelling affect how you feel about yourself? |  |  |  |  |
| Does the swelling affect your relationships with other people? |  |  |  |  |
| Does your lymphedema cause you pain? |  |  |  |  |
| Do you have any numbness in your swollen arm? |  |  |  |  |
| Do you have any ‘pins and needles’ or tingling in your swollen arm? |  |  |  |  |
| Does your swollen arm feel weak? |  |  |  |  |
| Does your swollen arm feel heavy? |  |  |  |  |
| Do you feel tired? |  |  |  |  |

| **9. In the past week** (Check one that best applies) | | | | |
| --- | --- | --- | --- | --- |
|  | Not at all | A little | Quite a bit | A lot |
| Have you had trouble sleeping? |  |  |  |  |
| Have you had difficulty concentrating on things, e.g., reading? |  |  |  |  |
| Have you felt tense? |  |  |  |  |
| Have you felt worried? |  |  |  |  |
| Have you felt irritable? |  |  |  |  |
| Have you felt depressed? |  |  |  |  |

**10. Overall how you would rate your quality of life at present?**  (Circle one number)

**0 1 2 3 4 5 6 7 8 9 10**

**Poor Excellent**

|  | Quality of Life Tool **LYMQOL – Lower Limb** | **Page 2** |
| --- | --- | --- |

**Do you have swelling in your lower limb?**  Yes No If no, tool is complete

| **11. How much does your swollen leg affect the following daily activities** (Check one that best applies) | | | | |
| --- | --- | --- | --- | --- |
|  | Not at all | A little | Quite a bit | A lot |
| Your walking |  |  |  |  |
| Your ability to bend, e.g. to tie shoelaces or cut toenails |  |  |  |  |
| Your ability to stand |  |  |  |  |
| Your ability to get up from a chair |  |  |  |  |
| Your occupation |  |  |  |  |
| Your ability to do housework |  |  |  |  |

| **12. How much does your swollen leg affect the following questions** (Check one that best applies) | | | | |
| --- | --- | --- | --- | --- |
|  | Not at all | A little | Quite a bit | A lot |
| How much does the swelling affect your leisure activities? |  |  |  |  |
| How much do you have to depend on other people? |  |  |  |  |
| How much do you feel it affects your appearance? |  |  |  |  |
| How much difficulty do you have finding clothes to fit? |  |  |  |  |
| How much difficulty do you have finding clothes you would like to wear? |  |  |  |  |
| How much difficulty in finding shoes to fit? |  |  |  |  |
| How much difficulty in finding socks/tights/stockings to fit? |  |  |  |  |
| Does the swelling affect how you feel about yourself? |  |  |  |  |
| Does the swelling affect your relationships with other people? |  |  |  |  |
| Does your lymphedema cause you pain? |  |  |  |  |
| Do you have any numbness in your swollen leg? |  |  |  |  |
| Do you have any ‘pins and needles’ or tingling in your swollen leg? |  |  |  |  |
| Does your swollen leg feel weak? |  |  |  |  |
| Does your swollen leg feel heavy? |  |  |  |  |

| **13. In the past week** (Check one that best applies) | | | | |
| --- | --- | --- | --- | --- |
|  | Not at all | A little | Quite a bit | A lot |
| Have you had trouble sleeping? |  |  |  |  |
| Have you had difficulty concentrating on things, e.g., reading? |  |  |  |  |
| Have you felt tense? |  |  |  |  |
| Have you felt worried? |  |  |  |  |
| Have you felt irritable? |  |  |  |  |
| Have you felt depressed? |  |  |  |  |

**14. Overall how you would rate your quality of life at present?**  (Circle one number)

**0 1 2 3 4 5 6 7 8 9 10**

**Poor Excellent**
